# Language Concordance and Interpreter Use in Primary Care: Perspectives from Spanish-preferring Patients

**DOI:** 10.1007/s10903-025-01768-w

**Published:** 2025-09-03

**Authors:** Zachary Predmore, Nabeel Qureshi, Mary E. Slaughter, Shannon Walsh, Yareliz Diaz, Efrain Talamantes, Yesenia Curiel, Rosa Elena Garcia, Denise D. Quigley

**Affiliations:** 1 RAND, Santa Monica, United States; 2 RAND, Boston, United States; 3 AltaMed, Los Angeles, United States

**Keywords:** Patient experience, Primary care providers, Quality of care, Spanish-preferring patients, Language concordance, Interpreter use

## Abstract

Healthcare provided by bilingual providers or with assistance from qualified interpreters is intended to improve patient-provider communication. Despite federal laws requiring healthcare facilities to provide access to appropriate interpretation language assistance services for patients not proficient in English, many Spanish-preferring patients receive primary care from providers not fluent in Spanish or who regularly use formal interpreters. Partnering with two urban SafetyNet providers in Southern California, we conducted focus groups in Spanish with Spanish-preferring patients who received care from providers who: (1) were Spanish-qualified, (2) used formal interpreters, and (3) used informal interpreters or other communication strategies. We coded transcripts to identify themes and compared patient experiences across provider types. Our study included 62 adult Spanish-preferring primary care patients. Spanish-preferring patients reported preference for continuity with their English-speaking providers despite language barriers because of established rapport. Patients receiving care from Spanish-language-qualified providers reported greater trust, more comprehensive care (i.e., covered more issues with minimal detail), yet with many interactions rushed. Formal interpreters facilitated better understanding and professional communication, however, impersonalized some patient-provider interactions. Informal interpreters or ad-hoc strategies led to mixed experiences, often dependent on patient or provider ability to accurately convey medical information. Overall, Spanish-preferring patient experiences highlighted the necessity for healthcare systems to support robust language and interpretation services that enhance direct communication, ensure interpreter quality, and maintain long-term patient-provider relationships. Improvements in policy and practice are needed to optimize healthcare communication for Spanish-preferring patients, since patient-provider communication is critical for high-quality health outcomes and experiences in multilingual settings.

**Supplementary Information:**

The online version contains supplementary material available at 10.1007/s10903-025-01768-w.

## Background

The U.S. Hispanic population is large and increasing, exceeding more than 62 million and accounting for most U.S. population growth since 2010 [1, 2]. Although 72% of Hispanics aged five and older speak English proficiently [2], many, particularly immigrants, do not.

The federal government requires federal agencies to ensure equal access to services and programs for individuals with limited English proficiency [3]. In particular, federally-funded healthcare providers, including Medicare or Medicaid providers, must provide qualified interpreters to patients with limited English skills [4]. Additionally, the National Standards for Culturally and Linguistically Appropriate Services (CLAS) in Health and Health Care highlight the need for trained interpreters to help eliminate disparities [5]. Yet there has been little progress in bridging communication and care quality gaps for patients who do not speak English [6].

Healthcare systems and outpatient clinics have several options, each with strengths and weaknesses, to serve Spanish-preferring patients. Bilingual-qualified healthcare providers can easily communicate with Spanish-preferring patients in Spanish, and research suggests patients are more comfortable discussing sensitive information with providers who speak their language [7]. Yet only 22% of U.S. family-care physicians are Spanish-speaking [8], and many need training in medical Spanish to be proficient in conducting healthcare encounters.

Providing interpretation is another common strategy for language support. Third-party interpreters, who are proficient in English and Spanish and can interpret accurately, can be present in a clinic or connected by phone or video. The California Healthcare Interpreting Association released standards in 2002 that highlight the role of interpreters as message converters, message clarifiers, cultural clarifiers, and sometimes patient advocates [9]. Most providers in outpatient clinics report having qualified, professional third-party interpreters available, though fewer than one-third use interpreters regularly [10]. Use of formal interpreters often poses technical difficulties (e.g., poor acoustics [11], issues with wireless internet [12] or phone reception). Formal interpretation may also change the interaction between patient and provider [13, 14] (e.g., increasing use of gestures [15], mediating emotional reactions through the interpreter [16], increasing outpatient visit length [17]), and impacting understanding of treatment and diagnoses [18]. Some providers rely on patient’s family or friends as informal interpreters [19, 20]. The CLAS standards discourage use of this practice [5], which some past research suggests may be used by practices to save money [21]; this approach raises concerns about patient confidentiality, accuracy of translation particularly for technical medical terms [22] and often results in miscommunication or misunderstandings that lead to misdiagnosis or other harm to patients [23]. Providers who speak some Spanish may “muddle through” the visit with a patient who can speak some English, using visual aids to help in their physical assessment and/or relying on translation apps (e.g., Google Translate) to bridge communication gaps [24–26]. While the impact of language concordance on healthcare quality and outcomes is well-documented [27–31], patient perspectives on language concordance could help identify areas for improvement both in practice and future federal guidance on maintaining high-quality provider-patient communication. To better understand patient experiences and perspectives on communication and language support strategies for Spanish-preferring patients, we conducted focus groups in Spanish with Spanish-preferring patients who received care from providers who: (1) were Spanish-language-qualified, (2) used formal interpreters, or (3) used informal interpreters or other communication strategies. We partnered with two large, urban Federally Qualified Health Centers (FQHC) with approximately 400 combined providers across 60 primary care clinics in two Southern California counties. Combined they had more than 1 million visits annually by patients who were predominantly Hispanic or Spanish-preferring. The FQHC tested providers to qualify them in Spanish using the Qualified Bilingual Staff assessment and the Clinician Cultural and Linguistic Assessment (CCLA). Providers were designated as Spanish-language-qualified if they scored above a 9 (on 1–12 scale) on the Qualified Bilingual Staff assessment and passed levels 1 and 2 on the CCLA. Per FQHC policy, only Spanish-language-qualified providers can communicate with Spanish-preferring patients without a formal interpreter present. For providers who are not Spanish-qualified, the FQHCs offered remote video or phone interpretation [32, 33]; they did not offer in-person interpretation. However, patients reported experiences where providers may not follow this policy and use informal interpreters such as nurses, staff, or family members, or manage with their own limited proficiency in 10.1007/s11606-025-09414-9 Spanish. Moreover, video interpretation was the default modality; phone was only used in rare cases when the patient was not comfortable with video or there were issues with the connection.

### Theoretical Framework

**Fig. 1:**
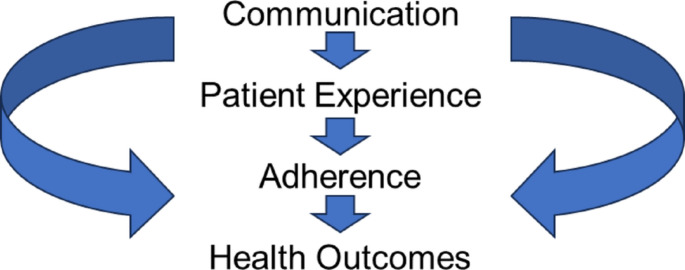
Theoretical Framework Adapted from [34]

We adapted an existing framework by Betancourt that had been previously developed using a systematic literature review addressing cultural competence in healthcare [34] that showed how provider-patient communication influences patient experience, which in turn influences adherence to treatment and, ultimately, health outcomes [34]. The framework originally had “Patient Satisfaction” where we have “Patient Experience” (Fig. 1).

## Methods

### Study Type

This is a cross-sectional descriptive, qualitative study using focus groups.

### Participants

**Fig. 2:**
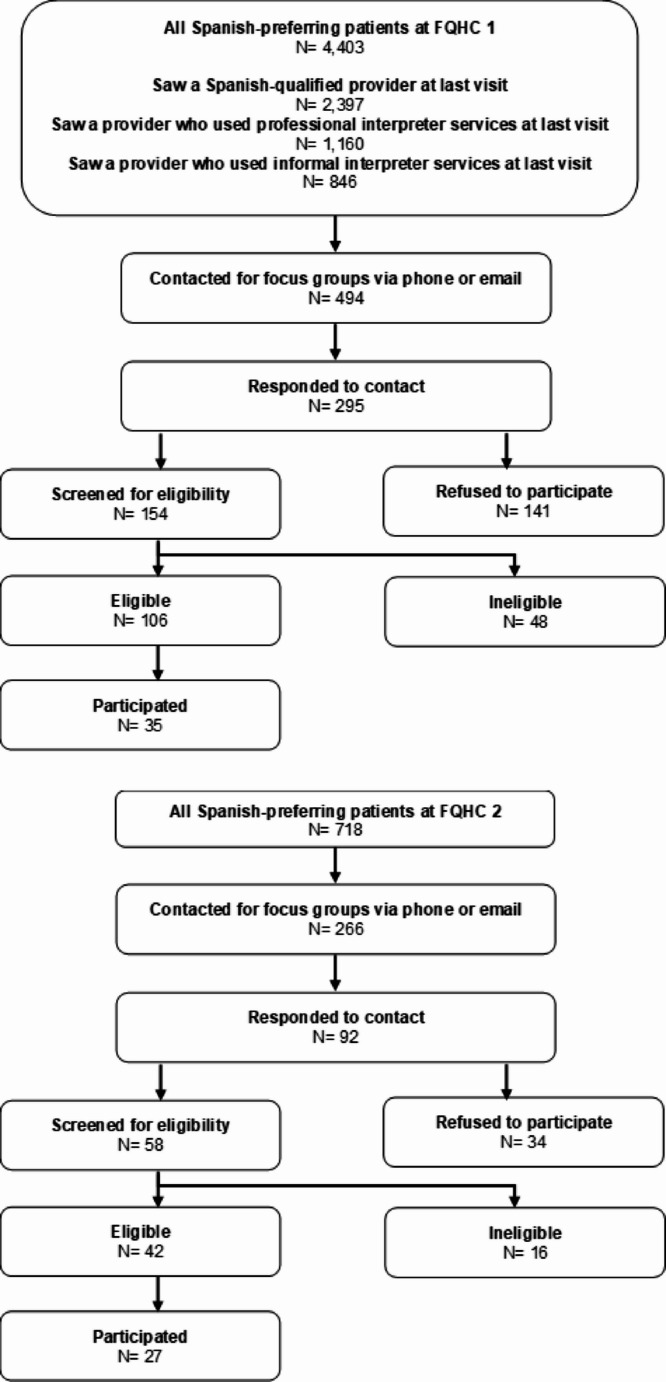
Sampling and Recruiting Flow Diagram

The FQHCs provided names, contact information, gender, date of last visit, and provider type (e.g., medical doctor, nurse practitioner, physician assistant) for all Spanish-preferring patients who had a face-to-face visit between September 15, 2022, and March 15, 2023 (FQHC#1: *n* = 4,403, FQHC#2: *n* = 718) (Fig. 2).

We stratified the data by provider type and created random sampling queues to recruit 6–8 participants per group. To be eligible, patients needed to be 18 or older, prefer to speak Spanish, and have been seen by the FQHC in-person at least once in the previous six months. We contacted patients by phone and screened patients to determine whether at their last visit their provider spoke Spanish, used a formal interpreter, or used another strategy (see Supplemental Materials for detail). We aimed for a balanced mix of participants by gender, age, nationality, and educational level in addition to having a mix by clinic site and specific provider.

### Data Collection

We conducted 9 in-person Spanish-language focus groups with Spanish-preferring patients from June 10 to August 10, 2023. These included three focus groups who last saw a Spanish-qualified provider (23 patients total), three groups who last saw a provider who used professional interpreters (phone or video) (24 patients total), and three groups who last saw a provider who used informal interpreters (15 patients total).

Each focus group lasted 90-minutes and was led by two bilingual team members using a structured discussion guide developed based on findings from a literature review on the relationship between patient experience and provider-patient language concordance [6]. The guides contained questions about topics including the way providers treated patients, any issues with communication during visits, experience with language support services, and opportunities for improvement with language issues. We recorded sessions with participants’ consent. Participants completed a demographic survey upon arrival (see Supplemental Materials for survey details) and were paid $120 honorarium.

We transcribed the audio-recordings and translated them using a professional transcription service. Bilingual members of the team reviewed and finalized the transcripts.

### Coding

We coded transcripts using Dedoose, a qualitative analysis program. We established codes based on the moderator guides and developed a code structure using systematic, inductive procedures to generate insights from responses [35, 36]. Four researchers participated in coding to identify topics, coding early transcripts independently and refining the codebook. After code training, we compared coding differences among coders and obtained a pooled kappa coefficient of 0.82, indicating “very good” coder agreement [37]. One researcher coded all remaining focus group transcripts and the other three researchers each coded two remaining transcripts so that all transcripts were coded by two coders. We used regular team meetings to reach consensus on topics, identify discrepancies, refine concepts, propose codebook changes, define codes, and discuss concepts and themes [38].

### Analysis

We conducted content [39] and thematic analysis [40] to identify themes. We compared the coded patient experiences for similarities and differences by participants who were language-concordant, used formal interpreters, or used another means of communication.

Study protocols were approved by RAND’s Human Subjects Protection Committee (IRB_Assurance No.:FWA00003425;IRB_No.:IRB00000051;Project ID:2023-N0018). All participants provided verbal informed consent prior to the focus groups.

## Results

### Patient Characteristics

Table 1 shows the characteristics of focus group participants. Most were 45 or older (82%), reported Mexico as their country of origin (79%), were female (55%), and had no hospital stay in the past year (60%). About half (47%) had graduated high school. Many reported health conditions such as high blood pressure (42%) or diabetes (29%). For the FQHC for which we had population data, patients participating in the focus groups reported similar scores on the CAHPS Clinician and Group Survey provider communication composite and overall provider rating at their last visit as compared to the FQHC’s Spanish-preferring patients during the same sampling window/timeframe (data not shown; p-value < 0.05).

**Table 1:** Participating Spanish-preferring patient characteristics

	Overall *N* = 62	
Characteristics	**Mean**	**SD**
Age	55 years	11
Age Groups	**N**	**%**
25–34	2	3%
35–44	9	15%
45–54	21	34%
55–64	16	26%
65–74	11	18%
75 or older	2	3%
Unknown	1	2%
Female	34	55%
Country of Origin		
Mexico	49	79%
Central American*	9	15%
South America	2	3%
Cuban	1	2%
Unknown	1	2%
Educational Attainment		
8th Grade or less	20	32%
Some High school	13	21%
High school Graduate/General education degree (GED)	20	32%
Some College	5	8%
4 year College Graduate or more	2	3%
Unknown	2	3%
Hospital Stay in the Past Year		
0 = None	37	60%
1 = 1 stay	19	31%
2 = 2 stays	2	3%
3 = 3 or more stays	1	2%
Unknown	3	5%
Ever Told Have Health Condition (Yes)		
Heart attack	1	2%
Heart disease	2	3%
High blood pressure	26	42%
Cancer	2	3%
Lung disease	2	3%
Asthma	9	15%
Diabetes	18	29%
Depression	11	18%
Self-Reported Overall General Health Status		
Excellent	4	6%
Very good	10	16%
Good	23	37%
Fair	21	34%
Poor	1	2%
Unknown	3	5%
Self-Reported Overall Mental Health Status		
Excellent	22	35%
Very good	14	23%
Good	16	26%
Fair	9	15%
Poor	1	2%
Type of Provider During Patient’s Last Visit at FQHC		
Doctor of Osteopathy/Medical Doctor	38	61%
Nurse Practitioner/Physician Assistant	24	39%
Last Visit Location Matched Patient’s Paneled Clinic**		
Visit location matched paneled clinic	31	89%
Visit location does NOT match paneled clinic	4	11%
Provider at Last Visit was Paneled Primary Care Provider**		
Provider at last visit was paneled primary care provider	20	57%
Provider at last visit was NOT paneled primary care provider	15	43%
Interpreter Service Rendered at Last Visit**		
Patient/Guardian refused interpreter services	9	26%
Provider spoke patient/guardian’s preferred language	18	51%
Staff interpreted	1	3%
Telephone Interpretation	2	6%
VRI (Video Remote Interpretation)	5	14%

NOTE: * includes El Salvador, Guatemala, Honduras, and Nicaragua. **We only had this information for patients at one of the FQHCs; SD = standard deviation

We identified two main themes: similarities noted by Spanish-preferring patients across provider and modality of interpretation, including preferences for continuity of providers (*Theme#1*), and differences in care experiences by provider communication strategy, including degree of trust in provider, comprehensiveness of visits, and degree of understanding (*Theme#*2).

*Theme#1*:*Spanish-preferring patients*,* despite the type of provider*,* did not want to change their provider*,* preferred the ability to speak directly to a provider*,* and utilized bilingual back-office staff to explain things in Spanish after seeing their provider.*

Table 2 provides illustrative quotes for Theme#1 in the original Spanish from the group discussion and translated into English. Spanish-preferring patients, regardless of current provider, consistently did not want to change providers. Most said they would rather deal with the known issues and challenges with their given provider who already knows them, their history, and their treatment. Patients with providers who used some form of interpretation were slightly more agreeable to changing providers, commonly due to the provider not having Spanish fluency or the patient wanting to talk directly to a provider rather than through an interpreter, but overall most did not want to change providers.

**Table 2:** Similarities in experiences for Spanish-preferring patients across provider types

Theme	English Quote	Spanish Quote
Patients did not want to change their providers	“I wouldn’t, because I think that when you have been with the same doctor for a long time, there is trust. You have the trust, the history, and you know he cares about your well-being, he makes sure you are doing as he told you, that is, he already knows you.” – ***Used informal interpreter – A3B3-Female***	“Yo no. Yo no porque pienso que cuando mucho tiempo estás o continúas con el (mismo) doctor hay esta confianza. Hay una confianza, hay historia. Hay una preocupación de él que usted esté bien, de que usted está haciendo lo que te dan, y ya me conoce.” – ***Used informal interpreter – A3B3-Female***
Patients preferred ability to speak directly with provider	“I had [a doctor] before who spoke perfect Spanish, and I felt that I could explain a little more to him, instead of talking to the interpreter and then the interpreter to the doctor. And so I felt more confident about what I was talking to the doctor, and I could talk to him even more.” – ***Used formal interpreter – A2A-3 Male***	“Antes tuve un [médico] que hablaba perfectamente español y me sentía un poco más podía explicar con él, más que cuando estoy hablando con el intérprete y luego el intérprete con el doctor, y así me sentí más seguro de lo que le estoy hablando con el doctor y puedo hablar más.” – ***Used formal interpreter – A2A-3 Male***
Patients expressed most trust in providers fluent in Spanish	“Because she has had my record for several years, she knows the good, the bad, the worst and the best things about my body and about everything. I trust her and she cares about me, I trust her to treat me; for example, I have the pap smear test done with her, so I trust that she knows everything. She knows that Latinas are very careful about the doctor who treats us, so you want to go to the doctor with trust, without her judging and prejudging you for things you have, you know? So, that’s the trust you have with that doctor.” – ***Spanish-Spanish language concordant – A1A-4 Female***	“Porque ya ella tiene todas mis registros de muchos años. Sabe lo bueno, lo malo, lo peor, lo mejor de mi cuerpo y de todo. Tengo confianza en ella y ella se preocupa por mí, confianza en que ella me trate. Por ejemplo, me hecho la prueba de Papanicolaou con ella, sí que confío en que ella lo sepa todo. Ella sabe que mujeres latinas tienen mucho cuidado con el doctor que nos trata, así quieres ir al doctor con confianza y sin que ella te juzgue y los prejuzgue por cosas que uno tiene. ¿Entiendes? Entonces, esa es la confianza que tienes con este doctor.” – ***Spanish-Spanish language concordant – A1A-4 Female***
Bilingual back-office staff play important role by explaining things in Spanish after seeing provider	“[The providers] ask whether I understood. And as with everything, there are nurses who are very nice people, and they have the patience to explain things.” – ***Used informal interpreter – A3B-3 Female***	“[Ellos] me preguntan de que yo entiendo y, pues con todos, hay enfermeras que son muy buenas personas y que tienen paciencia para explicar cosas.” – ***Used informal interpreter – A3B-3 Female***

All Spanish-preferring patients said being able to speak directly to a provider or through a formal interpreter makes it easier for them to understand what is happening during the visit and makes them feel more comfortable. Patients expressed the most trust in providers who are fluent (i.e., Qualified) in Spanish.

Spanish-preferring patients seen by all three types of providers said that back-office staff provided additional clarity or explanation in Spanish after patients met with the provider to ensure they understood next steps, treatment or diagnoses. In most cases this occurred when the provider needed to use an interpreter but did not or relied on their limited grasp of Spanish.

*Theme#2*:*Spanish-preferring patients have qualitatively different experiences when seen by a language-concordant provider or when using an interpreter (formal or informal).*

Tables 3, 4 and 5 provide illustrative quotes in Spanish and English for Theme#2 by provider type.

**Table 3:** Illustrative quotes of experiences of Spanish-Preferring patients receiving care from a Language-concordant provider

Theme	English Translation of Quote	Quote in its Original Spanish
Providers reviewed all issues that the patient had with the patient	“The doctor is really kind and always checks my lungs, my heart, he takes the time to explain to me what we are going to do, step by step; he explains the results of the analyses or the tests perfectly. When I went to this clinic, I didn’t know everything I had, I just felt depressed. Once in the clinic, they diagnosed me with ulcerative colitis and irritable bowel syndrome, so I’m on a diet and taking medication; the doctor always sees what medicines I can take for all the health problems that I have, but he is really good.” – ***Spanish-Spanish language concordant – A1A-7 Female***	“El doctor es muy amable y me revisa siempre los pulmones, el corazón, se toma el tiempo de explicarme lo que vamos a hacer paso a paso. Le explica los resultados perfectamente de los analisis o los estudios que haya hecho. Cuando llegué a esta clínica, no sabía todo lo que yo tenía, solo sentía que tenía depresión. Una vez estando en la clínica, me diagnosticaron colitis ulcerosa e intestino inflamables, entonces estoy en una dieta y estoy… tomo medicina. Ellos siempre anda viendo que medicinas puedo tomar para todos los problemas de salud que tengo, pero él es muy bueno.” – ***Spanish-Spanish language concordant – A1A-7 Female***
Providers discussed preventative health, diet, and exercise	“Yes, also, walking to help with fatty liver or things like that. Exercising more. Sometimes, she gives me medication to lower my cholesterol, if my cholesterol isn’t going down for whatever reason. Things like that. […] Yes, she’s always on top of all that.” – ***Spanish-Spanish language concordant – A1B-8 Male***	“Sí, también caminar para ayudar con el hígado graso o cosas así. Más ejercicio. Me da medicamentos a veces para bajar mi colesterol, si por algo cosas mi colesterol no baja. Cosas como eso. […] Sí, ella siempre está entienden de todos esos.” – ***Spanish-Spanish language concordant – A1B-8 Male***
Providers tell the patient information verbally and in writing	“I also get everything in writing and in Spanish, that’s how I understand it and it has solved many things. I’m dealing with the disease and my health has improved a lot” – ***Spanish-Spanish language concordant – A1A-6 Male***	“A mí también me da todo estopor escrito todo en español a mi manera de entender y, y pues me ha solucionado muchas cosas. Estoy lidiando con enfermedad y ya me he mejorado mucho mi salud.” – ***Spanish-Spanish language concordant – A1A-6 Male***
Providers provided bilingual written information to ensure mutual understanding	“In my case, yes, that is, always at the end of each consultation, they always give me, let’s say, a summary of the appointment, right? And in my case, I am also given information about the discomfort in my knees. They say, do these exercises at home, it’s written very well, it is mostly written in Spanish. And there is very little information that cannot be translated into Spanish, and they write it in English.” – ***Spanish-Spanish language concordant – C1C-6 Male***	“En mi caso, sí, o sea, siempre al final de cada consulta me dan, digamos, un resumen de la cita, ¿no? Y en mi caso también me dan información sobre las molestias en mis rodillas. Se me dice haz esto ejercicio en casa. Escrito muy bien, escrito en su mayoría en español. Y hay muy poca información que no puede ser traducida al español y escriben en inglés.” – ***Spanish-Spanish language concordant – C1C-6 Male***
Providers followed up after the visit	“Yes. She even asked me—I was sent for an ultrasound before, where they had supposedly found something. I say, “supposedly,” because I don’t see that I have anything. So, she asked me, “Did you ever get a follow up on your checkup, the ultrasound?” I was like, “No, it’s always the same. I don’t feel bad. I feel really good.” She said, “Go there again.” She wanted to follow up. So, I went to do the ultrasound again, and she called me on the phone to tell me that the results were back and that everything was fine. There was nothing to worry about. So, it was a good experience.” – ***Spanish-Spanish language concordant – A1B-12 Female***	“Si, incluso allí me preguntó – donde yo antes, me mandó a un ultrasonido, que me encontró algo allí, según yo digo porque yo no miro nada. Y entonces me dijo “¿cómo se siguió su siguiente chequeo?” Y dije, “no pues siempre lo mismo, no me siento mal, me siento muy bien,” y dice, “vaya otra vez, hágaselo otra vez.” Me da seguimiento. Fui a hacérmelo el ultrasonido y me llamó por teléfono, para decirme, que ya están los resultados, que salieron bien, que no hay nada de qué preocuparse. Sí, fue una buena experiencia.” – ***Spanish-Spanish language concordant – A1B-12 Female***
Patients trusted their providers	“To me, the doctor has been a good friend. When we talk about my disease and some things that are happening to me, we don’t talk, we understand each other. He is really helpful, because there are some doctors that tell you off and you get nervous. So, my current doctor is like a friend who cares about me, who says, “take your meds,” so you take care of yourself, that’s what I like about my doctor.” – ***Spanish-Spanish language concordant – A1A-8 Male***	“Pues a mí el doctor ha sido un buen amigo cuando platicamos de mi enfermedad o algo que me pasa. No platicamos, nos entendemos. Él me ayuda mucho, porque hay doctores que le llaman la atención a uno y se pone uno todo nervioso de ahí. Entonces el doctor ahorita es como un amigo, “mira cuídate, toma tus medicinas”, entonces solito uno se va cuidando. Eso es lo que me gusta del doctor.” – ***Spanish-Spanish language concordant – A1A-8 Male***
Providers ensured understanding	“In my case he asks me and says, ‘did you understand everything correctly? Do you have any questions for me? Something you didn’t understand?’ He always makes sure I understood everything well and if I understood medications […] In my case, he uses other words, other synonyms, more or less so that I can understand and he makes sure that I am understanding. Sometimes I know that because of their vocabulary, their medical training, they use it instinctively, but then he says, ‘do you know what that is?’ And I say, ‘yes,’ or I say, ‘no,’ and he looks for other words.” – ***Spanish-Spanish language concordant – C1C-8 Male***	“En mi caso, me pregunta y me dice, “¿Entendiste todo bien? ¿Tienes alguna pregunta para mí, ¿algo que no entiendes?” Él siempre se asegura de que yo entienda a todo bueno y si entiendo medicaciones […] En mi caso, usa otras palabras, otros sinónimos, más o menos para que yo pueda entender y se asegura de que estoy entendiendo. A veces, sé que por su vocabulario, por su formación médica, le usan intuitivo, pero luego dice, “¿si sabes lo que es?” Y digo, “sí,” o digo, “no,” y busca otras palabras.” – ***Spanish-Spanish language concordant – C1C-8 Male***
Providers asked open-ended questions	“My doctor in [FQHC #2] is really good. Just like all of you said, “How do you feel? How are you?” He devotes all his attention to me, he talks to me, he assists me well, he greets me well and I can explain everything I feel, everything I have to him, the medicines I take, and he pays full attention to me.” – ***Spanish-Spanish language concordant – A1A-9 Female***	“En [FQHC #2], el doctor que tengo es muy bueno. Igual como los escuché a todos, “¿cómo te sientes?, ¿cómo estás?” Me pone toda la atención, me habla, me atiende bien, me saluda bien, y yo puedo explicar todo lo que siento, todo lo que tengo a él, las medicinas que tomo, y él me pone toda atención.” – ***Spanish-Spanish language concordant – A1A-9 Female***

**Table 4:** Illustrative quotes of experiences of Spanish-preferring patients receiving care from providers using formal interpreters

Theme	English Translation of Quote	Quote in its Original Spanish
**Impact of formal interpretation on provider communication**		
Providers were professional, attentive, respectful, and punctual	“This is the second time that I’ve gone to the [1st CLINIC]. I’ve always gone to the [2nd CLINIC]. This was my second visit to the clinic I liked it. I felt comfortable and at ease with the doctor. It was my second time seeing her. She was very professional. She took care of me and listened to me.” – ***Used formal interpreter – C2B-7 Female*** “I was there in March due to an accident, and a doctor assisted me. She didn’t speak Spanish, and they used the translator service. It went really well. The lady was very professional. I mean, both the doctor and the translator were really good. I felt well, really comfortable, and they took very good care of me.” – ***Used formal interpreter – 2B-1 Male*** “I always try to be there a few minutes early for the consultation, because I know that you have to register, and then they tell you, ‘take a seat, we’ll call you soon’[…] The doctor was very punctual…he understood everything about my health problem[…] the doctor checked me, asked me some questions, which I responded perfectly[…] He prescribed me some medication and set up my next appointment, so as to continue with my treatment. He said I could do that over the phone, so that I didn’t have to take time off work and so on. I said, ‘okay.’ I can’t complain about the service, honestly.” – ***Used formal interpreter – 3B-5 Male***	“Esta es segunda vez que voy a la [1st CLINIC]. Siempre fui a la [2nd CLINIC]. Esta fue mi segunda visita a la clínica, me gustó. Me sentí cómoda y a gusto con la doctora. Era mi segunda vez que la veía. Muy profesional ella. Me cuidó y me escuchó.” – ***Used formal interpreter – C2B-7 Female*** “Yo estuve en marzo porque (tuve) un accidente y este me atiende una doctora. No hablaba español y utilizaron el servicio de traductores. Fue muy bien. Muy profesional la señora. Si tanto, la doctora como la traductora fue muy bien. Me sentí bien, muy cómodo, y me atienden muy bien.” – ***Used formal interpreter – 2B-1 Male*** “Siempre trato de llegar unos minutos antes de la consulta porque yo sé que tienes que registrarte, y luego te dicen, ‘toma asiento, vamos a llamar pronto.’ […] Fue muy puntual el doctor… Me entendió todo sobre mi problema de salud[…] Este doctor me chequeó, me hizo algunas preguntas, las cuales respondí perfectamente[…] Me recetó unos medicamentos y él me registró mi próxima cita para continuar con mi tratamiento. Dijo que podía hacerlo por teléfono para no tener que ausentarme del trabajo y demás. Dije, “está bien.” Honestamente, no puedo quejarme del servicio.” – ***Used formal interpreter – 3B-5 Male***
Providers focused on main reason for visit and used follow-up appointments for other issues/concerns	“And in my case, I don’t know if they experienced something similar, but you can’t ask anything other than the topic of your consultation. Because they only say, ‘no, this appointment is just to check this, not for this other thing. If you want to check that, get another appointment.’” – ***Used formal interpreter – C2C-3 Female***	“Y en mi caso, no sé si la experiencia de ellos es algo similar, pero no se puede preguntar difrente de que es más que el tema de tu consulta. Porque solo dicen, ‘no, tiene esta consulta solo para chequar esto, no es para eso. Si quieres para esto, entonces pasa nueva cita.‘” – ***Used formal interpreter – C2C-3 Female***
Using lots of gestures, pointing, and short sentences to improve communication	“Normally, when we speak Spanish, we also have some words that aren’t used in English. Sometimes, we say, ‘Oh, my tailbone hurts.’ The interpreter asks, ‘What’s that?’ So, they ask you exactly where you have the pain. And you have to explain exactly where the pain in. Because sometimes, they get confused when it’s chest pain, and you have to tell them exactly what hurts, so they can explain it well to the doctor. Because like I said, sometimes, we say, ‘My chest hurts,’ but it might actually be this [pointing to breast] that we’re calling our chest, but it might be our breast so sometimes we have to point or tell them. And before the interpreter tells the doctor they ask, ‘exactly where is the pain?’ So, we have to explain it, so that the interpreter can tell the doctor exactly where you’re having that pain.” – ***Used formal interpreter – 2B-4 Female*** “I have noticed that she tries to speak in shorter and easier phrases. Because if she talks a lot or with really long sentences, a lot of times, I think even the interpreter has trouble understanding.” – ***Used formal interpreter – C2B-7 Female***	“Normalmente, cuando hablamos español, tenemos unas palabras que no se usan en inglés. A veces nosotros decimos, ‘Oh, me duele la rabadilla.’ Y le dice el intérprete, ‘¿eso qué es?’ Entonces pregunta exactamente donde tienes dolor. Y hay que explicarse exactamente donde tienes el dolor. Porque a veces se confunden cuando es dolor en el pecho y hay que decirles exactamente que les duele, para que se lo expliquen bien al doctor. Porque, como dije, a veces nosotros decimos, ‘me duele el pecho.’ Pero en realidad podría ser esto [señala el seno] que llamamos nuestro pecho, pero podría ser nuestro seno, así que a veces tenemos que señalarles o decírselo. Y antes de que el intérprete se lo diga al doctor, le preguntan, ‘¿dónde está exactamente el dolor?’ Entonces tenemos que explicarlo, para que el intérprete pueda decirle al doctor exactamente donde sientes ese dolor.” – ***Used formal interpreter – 2B-4 Female*** “He notado que ella trata de hablar como frases más cortas y como más fáciles. Porque si ella habla mucho o en frases muy largas, muchas veces, pienso que hasta al intérprete le cuesta entender.” – ***Used formal interpreter – C2B-7 Female***
Many patients did not like working through third-party, as they felt uncomfortable discussing private health issues	“I’ve learned over time that although I’m embarrassed or ashamed, I have to speak up, because I’m not going home with that pain or that problem, because I’m there to seek help. So, I’ve had to put my shame aside, and speak with the interpreter.” – ***Used formal interpreter – C2C-3 Female***	“En mi caso, he aprendido que he sido más abierto con el tiempo, aunque a mí me de pena o me de vergüenza, tengo que hablar porque no me voy ir con el dolor, no me voy ir con el dolor o con el problema para la casa. Voy a buscar ayuda entonces. He hecho a un lado la vergüenza y he tenido que hablar con el intérprete.” – ***Used formal interpreter – C2C-3 Female***
**Interpreter quality varied**		
Interpreters translated everything, repeated and explained what was said, asked for clarification, and clarified what was said	“Yes, as I said at the beginning, when they are about to finish the consultation, the doctor, in my case, she makes sure by asking me, ‘did you understand that?’ Or ‘do you have any questions?’ So the interpreter says, ‘the doctor is asking whether you understood what she said,’ or ‘this is a good time for you to ask her.’ And if I ever have a doubt, I tell her, ‘listen, I’m not sure about this, could you repeat it?’ And I’ve seen that my doctor gladly repeats it.” – ***Used formal interpreter – C2C-3 Female***	“Sí, como le diga al principio, a cuando ellos están por terminar la consulta, la doctora, en mi caso, se asegura, me dice hacen pregunta conmigo, ‘¿entiendes eso?’ o, ‘tienes alguna pregunta?’ Y entonces el intérprete me dice, ‘la doctora le pregunta si entiendo lo que le dijo,’ o ‘es un buen momento para que le pregunte.’ Y sigue si yo tengo alguna duda le digo, ‘escucha, no estoy muy claro por eso, ¿por favor lo repites?’ Y he visto que lo repite con mucho gusto la doctora.” – ***Used formal interpreter – C2C-3 Female***
Interpreters increased patient confidence in talking to providers by asking providers to talk slower	“The interpreter tells the doctor to speak a little slowly so that he can interpret for us.” – ***Used formal interpreter – A2A-4 Female***	“El intérprete le dice al médico que hable un poquito más despacio para que el pueda interpretarnos a nosotros.” – ***Used formal interpreter – A2A-4 Female***
Interactions with interpreters sometimes included misinformation and misunderstandings	“My doctor always asks me if I have any questions, through the interpreter. And when the doctor tells him something, sometimes the interpreter misunderstands exactly what he’s trying to say, and he asks the doctor again, ‘are you saying this?’ And the doctor explains it better, so the interpreter understands and then says, ‘it’s this and this,’ and the doctor says, ‘correct,’ And then the interpreter tells me what it is, exactly what it is.” – ***Used formal interpreter – C2B-4 Female***	“Mi doctor siempre pregunta si tengo alguna duda incluso el intérprete. Cuando el doctor le dice algo, a veces el intérprete también no lo entiende bien exactamente lo que está queriendo decir y él le vuelve a preguntar al doctor ‘¿le estas diciendo esto?’ y el doctor le explica mejor lo que tiene que decir, entonces y ya después es okay ‘es esto y esto’ y la doctora le dice ‘correcto.’ Entonces ya el intérprete me dice lo que es, exactamente lo que es.” – ***Used formal interpreter – C2B-4 Female***
Patients with more English ability opted not to use an interpreter for sensitive issues	“It happened to me in the month of November that I spoke (about) something private, and I liked it because I didn’t say to the doctor something that I didn’t want the person to hear. But since I had already told the nurse, I told her to put it there so that we wouldn’t forget it in the middle of the consultation. She put it right there, clearly, so he told the interpreter that he was going to lower the volume. He said, “Oh, I’m sorry but I’m going to turn the volume down, because there is something here that I need…” And he turned it over.” – ***Used formal interpreter – A2A-21 Female***	“A mí me pasó en el mes de noviembre este si yo hablé algo privado y me gustó porque este yo no le digo nada al doctor que no quería que oyera la persona. Y entonces pero como ya le había dicho a la enfermera y le dije que lo pusiera ahí para que no se nos olvidara en medio de la consulta. Ella lo puso clarito entonces éste le dijo al intérprete que iba a bajar el volumen. Le dijo, “oh, I’m sorry pero voy a bajar el volumen, porque aquí hay algo que necesito” y le dio la vuelta.” – ***Used formal interpreter – A2A-21 Female***

**Table 5:** Illustrative quotes of experiences of Spanish-preferring patients receiving care from providers using informal interpreters

Theme	English Translation of Quote	Quote in its Original Spanish
**Impact of informal interpretation on communication**		
Providers showed more empathy	“One of the things I liked about him was that he helped me by telling me not to worry, that everything was going to be fine, about what they had found in my lung, the spot they found in my lung. He said, ‘Don’t worry, everything is going to be fine.’ Because in those days I was a little bit upset, worried because I was wondering, ‘Is it going to be cancer?’ And I was not sleeping well, and I had a lot of headaches. So I explained to him that I had a lot of headache and that I was not sleeping well, and he gave me a lot of encouragement. ‘Don’t worry,’ he said, ‘everything is going to be fine.’” – ***Used informal interpreter – C3A4-Male***	“Una de las cosas que me gusto de él, que me ayudó decirme cosas que no me preocupara que iba salir todo bien, de lo que me había encontrado en el pulmón. De la mancha que me encontraron en el pulmón. Dice ‘no te preocupes’, dice éste, ‘todo va salir bien, no te preocupes,’ porque yo en esos días andaba un poco alterado, estaba pensando, ‘¿va ser cáncer?’ Y usted sabe de que uno se lo mete en la cabeza y lo trae trabajando como ahí. Y no dormía bien, y me dolía mucho mi cabeza. Entonces este le explique a él que tenía mucho dolor de cabeza y que no dormía bien y él me dio mucho aliento decirme que, ‘don’t worry,’ dice ‘todo va a salir muy bien.’” – ***Used informal interpreter – C3A4-Male***
Providers spoke slowly	“What I do is try not to let him talk to me too fast. If I don’t catch what he’s saying, I tell him to go back and speak more slowly. And that’s the system I have.” – ***Used informal interpreter – A3C-3 Male***	“Lo que hago yo trato de que no me hable muy rápido. Si no capto lo que está diciendo, le digo que vuelva atrás y que hable más despacio. Y ese es mi sistema que tengo.” – ***Used informal interpreter – A3C-3 Male***
Providers spent more time with patient to ensure mutual understanding	“So they give you time, they listen to us, and then they explain things that are coming up for the next visits. And if there are referrals to other doctors or if there are any different concerns, they also explain to us the whys, and I think that’s the way it should be.” – ***Used informal interpreter – C3A-1 Male***	“ahora nos dan tiempo, nos escuchan, y luego nos explican las cosas que van a venir para las siguientes visitas. Y si hay referencias a otros doctores o si hay alguna preocupación diferente también nos explican los porques y es así pienso que es lo que debería ser.” – ***Used informal interpreter – C3A-1 Male***
Providers use some Spanish to aid communication	“I mainly refer to a doctor who is my main doctor, and we have a system that I speak English and Spanish with her, because she is very accessible to both languages. Sure, she speaks better English, but she is very accessible in the Spanish language as well, she likes to speak it, and I feel at ease.” – ***Used informal interpreter – A3C-7 Male***	“Yo principalmente me refiero a una doctora que es mi doctora principal y tenemos un sistema de qué con ella, hablo inglés y hablo español. Porque es muy accesible a los dos idiomas. Claro, habla más inglés, pero es muy accesible a su idioma de español, le gusta hablarlo y yo me siento agusto.” – ***Used informal interpreter – A3C-7 Male***
**Reasons for not using an interpreter in these visits**		
Providers did not ask if patients wanted to use an interpreter	“The doctor treated me very well, except that she didn’t ask me if I wanted an interpreter, when it is an obligation for her when you are a Latino. And I felt a little uncomfortable because, besides the fact that I was in a hurry, I explained my problem as best I could. She understood me, but I would say that I had to be a little more professional and say ‘okay, are you [proper noun] Latino?’ Besides, she is my doctor and I have used an interpreter before. So, this time, she didn’t ask me.” – ***Used informal interpreter –*** **C3A-4 Male**	“La doctora me trató muy bien, excepto que no me preguntó si quería intérprete, cuando es una obligación de ella cuando es como Latino. Y me sentí un poco incómodo porque, aparte de que llevaba prisa, le expliqué mi problema lo mejor que pude. Me entendió y todavía que tenía que ser un poco más profesional que ella y decir, ‘okay, ¿usted es latino?’ Aparte, ella es mi doctora y he utilizado a la intérprete. Entonces en esta vez ella no me preguntó.” – ***Used informal interpreter – C3A-4 Male***
Patients felt the issues to be discussed were not important enough to use an interpreter	“He asked me if we could use the nurse as an interpreter, and I told him no, because it was a minor thing to treat; just to make the appointments and that’s it. It wasn’t telling him where it hurt, which he already knows because he is treating me.” – ***Used informal interpreter – A3C-1 Male***	“También me preguntó él que si podíamos usar como interprete a la enfermera, yo le dije que no porque pues era algo leve de tratar; solo hacer las citas. Decirle donde me dolía, y él ya sabe porque el me está tratando.” – ***Used informal interpreter – A3C-1 Male***
Patients had an experience with a low-quality interpreter in the past	“In my case, I found that what I wanted to say was not really what the interpreter was translating. […] And I will tell you why. Because many times the interpreters are from South America, let’s say from Argentina, from further south, and we, Latin Americans from this side, use very different words. So they do not understand what you are saying but they are not communicating to the doctor what you mean either.” – ***Used informal interpreter – C3A-1 Male***	“En mi caso, yo encontré que lo que quería decir no era realmente lo que el intérprete está traduciendo […] Y le voy a decir por qué. Porque muchas veces los intérpretes son de sudamérica, digamos de Argentina, de más del sur, y nosotros, latinoamericanos de este lado, usamos palabras muy diferentes. Entonces no entienden lo que (uno está) diciendo pero no comunican al doctor lo que quieres decir.” – ***Used informal interpreter – C3A-1 Male***
Patients were uncomfortable with a third party in the visit	“I, personally, [not using an interpreter] has occurred during prostate exams. I’ll be honest with you, that’s it. I have tried my best to practically do it with the doctor, directly. And he does not speak Spanish.” – ***Used informal interpreter – A3C-7 Male***	“Yo, personalmente, [sin utilizar intérprete] me ha pasado por el chequeo [de] la próstata. Prácticamente seré honesto contigo, eso es todo. Yo he tratado lo mejor posible prácticamente hacerlo con el doctor directamente y él no habla español.” – ***Used informal interpreter – A3C-7 Male***

#### Language-concordant Experiences

Spanish-preferring patients reported that care experiences with a Spanish-qualified provider were “professional”/“profesional”, “cordial”/”cordial”, and respectful”/”respetuosa” and covered more issues but with minimal details (See Table 3). Patients indicated that language-concordant providers were more likely to explain things both verbally and in writing; provide written materials in a language other than English; discuss preventative care, diet, and exercise; treat them well; review all issues raised; ask open-ended questions; and follow-up after the visit. However, they reported that these providers less often listened to them or took the time needed to spend with them. In most cases, patients had long-term relationships with their provider.

No patient reported issues communicating with their provider, but a few patients said their interactions were rushed or limited in the amount of time spent with the provider. This could relate to general pressure among primary care physicians to see patients quickly; previous survey research has suggested that many patients report their doctors rush through exams [41]. One patient acknowledged this by saying doctors “have a lot of patients and the faster they get through with one, well, the better it is for them to move forward. And I agree that there are a lot of patients; it’s not just me. So one of the things I don’t like about the clinic here […] is that the doctors are always in a hurry.”/“Tienen muchos pacientes y más rápido terminen con uno, pues, es mucho mejor para ellos para advancar. Y estoy de acuerdo en que hay muchos pacientes, no sólo yo. Entonces una de las cosas que no me gustaba de la clínica aquí[.] es que los médicos siempre tienen prisa.”

Patients reported more trust with language-concordant providers (“[the provider] treated me really well and I trusted him, because we spoke Spanish so we had the trust to talk about everything/El proveedor me trató muy bien y confié en él, porque hablábamos español así que teníamos la confianza para hablar de todo”) but were hesitant or reluctant to bring up challenges (such as not being provided enough detail about issues discussed) in the visit. Patients indicated that language concordance was helpful for understanding, privacy, and comfort, both physical and emotional. Patients said staff helped them write down items such as medication names or steps needed as follow-up in Spanish.

#### Experiences Using Formal Interpreters

Patients with an English-speaking provider using formal interpretation reported that providers were “professional”/“profesional”, “attentive”/”atenta”, “respectful”/”respetuosa”, and “punctual”/”puntual” (See Table 4). Such encounters focused on the main reason for the visit and did not, because of interpretation time, have time to cover other topics, resulting in fewer issues being addressed. Nevertheless, such patients said the provider addressed their main reason for their visit well. They added that their needs were addressed by the provider, just not in one visit. Patients also reported that using an interpreter made visits more impersonal.

Patients noted that interpreters often spoke different dialects of Spanish, which hindered their understanding. Still, they said that using an interpreter improved understanding overall and increased their confidence in communicating with their provider. If the interpreter did not understand a term the patient used, the interpreter asked for clarification and ensured the patient understood what they were saying. Patients using a formal interpreter indicated that the greatest value of an interpreter was improving understanding between the patient and provider, including better communication with the provider, exact translations, and more explanations. Patients said interpreters were helpful because they clarified what doctors meant and asked the provider to slow down when speaking, which some patients were not comfortable doing. Patients with formal interpreters reported more mutual understanding across critical aspects of communication, including listening, spending time, and explaining. Such patients considered their providers professional and reported that their providers used gestures, pointing, shorter sentences, shared needed information, explained answers to questions, knew their medical history, discussed personal matters, and made them feel comfortable.

Some patients did not like working through a third-party as it made them uncomfortable or self-conscious with an additional person in the room, especially when addressing sensitive topics. They also reported misinformation and misunderstandings. Patients who understood some English often would opt to not use an interpreter, especially for routine visits. Office staff supported language and communication before the visit by asking the patient if they wanted an interpreter, or preferred Spanish as well as being available after the visit to clarify anything the provider said, explain things in Spanish, and set up future appointments.

#### Experiences Using Informal Interpreters

Patients reported that experiences with providers who use either informal interpreters or their own Spanish abilities were most often routine or follow-up visits to cover “simple things”/”Cosas sencillas” not important enough to ask for an interpreter (See Table 5). For “more complex”/”muy compleja” issues, most had seen a provider who used an interpreter. Patients reported challenges in visits using informal interpreters, primarily not always being asked about the main reason for the visit and not feeling that their needs were met. For example, one patient noted feeling “humiliated”/humillación” because the provider talked down to him; the patient felt the provider did not allow him to express himself and that the provider was rushing. Patients who saw providers using informal interpretation or other means of communication reported that these providers asked more open-ended questions, explained answers to questions including medical terms, provided needed information, knew and reviewed medical history, and asked if the patient had additional questions, but were not as consistent as those who were language-concordant or used formal interpreters. Patients with providers who used informal means to communicate reported that their provider spent longer time with them, spoke slowly, and explained things in different ways. These patients also voiced more often that these providers cared about them, showed empathy, and spent more time to ensure mutual understanding, and that they opened up more to these providers. Patients did say not having an interpreter available made them feel uncomfortable asking questions. The main reason that they did not use an interpreter was because they were not asked if they needed one.

## Discussion

Spanish-preferring patients across provider types almost all preferred to continue with their current providers despite any communication barriers. They valued their providers’ familiarity and understanding of their medical history, suggesting a significant reliance on established rapport over better language concordance or more consistent use of interpreters. This suggests that patients are unlikely switch providers to improve communication, and efforts to improve language support need to come from providers and health systems. Providers should be aware of these results and can use them as the basis for starting conversations about communication with their Spanish-preferring patients.

Patients reported that with Spanish-qualified providers they felt greater trust, received professional care that was more comprehensive yet with many interactions being rushed. Patients with providers who used formal interpreters reported that they appreciated the professional demeanor and clear communication interpreters facilitated, because interpreters helped bridge the language gap and enhance their understanding despite making the visits longer and feeling somewhat impersonal. However, these encounters also had challenges, including Spanish dialect differences and adequacy of interpretation.

Patients with providers who used other means of communication reported mixed experiences. While some patients felt more at ease and perceived a greater degree of empathy and effort to understand their needs, others reported that they faced significant communication barriers that hindered the effectiveness of their visits.

Our findings suggest that Spanish-preferring patients receiving primary care through urban SafetyNet providers have opted into the communication strategy that they prefer. The preference for care continuity that patients reported underscores the need for healthcare systems to support and maintain long-term patient-provider relationships, which are foundational to patient trust, communication, overall care experience, and retention. Still, the ability for Spanish-preferring patients to communicate directly with their providers, whether through language concordance or effective interpretation, significantly enhances their comfort and understanding during visits.

Spanish-preferring patients also reported challenges centered around language barriers, quality and consistency of interpreters, and the nature of their interactions with providers. Key issues included developing trust with non-Spanish speaking providers, mixed experiences with using formal interpreters, and the impersonal nature of having an interpreter that made discussing sensitive health issues difficult. Patients expressed concern over rushed communications with providers, even those fluent in Spanish, and not being proactively offered interpretation services. Our finding of Spanish-preferring patients expressing concerns about being rushed in outpatient care visits is consistent with evidence that many patients (including Spanish-preferring and English-speaking patients) feel rushed in outpatient care visits [41]. Previous research has found providers sometimes do not use interpreters due to time constraints [42], so FQHCs and other clinics could schedule longer visits when a patient plans to use an interpreter to allow for all the patient’s concerns to be addressed.

Our study has limitations. While the sample is relatively large for qualitative inquiry, the experiences of our participants may not reflect the experiences of Spanish-preferring patients receiving primary care in a SafetyNet setting nationwide. Additionally, the focus-group moderator may have unknowingly guided or influenced the conversation; however, our selection of a moderator with significant experience conducting focus groups on healthcare topics in English and Spanish limited this potential bias. Focus group dynamics may bias certain participants into agreeing with others; however, our moderator at the beginning of each group laid out clear expectations about our interest in each participant voicing their different opinions and experiences and made sure to collect input from all participants during discussions. Lastly, we coded and analyzed the transcripts in English, which may have resulted in loss of nuance of what participants said in Spanish; however, we had the bilingual moderator and another bilingual team member review the English transcripts to ensure accuracy. We also included quotes in English and Spanish to provide the original language and meaning.

There are several considerations for future researchers to explore the impact of language concordance and communication modality on primary care. Patients may view providers who learned Spanish and become qualified differently from providers who are native Spanish speakers. Future researchers should take these potential differences into account when designing studies. We also did not probe for major differences between video and phone interpretation. Future studies could explore the differences between using interpreters via video, in which the interpreter can communicate non-verbally, and via phone, in which the interpreter cannot, to better understand the patient perspective on these interpretation scenarios.

Our findings indicate the need for strategies to enhance communication between providers and Spanish-preferring patients regardless of provider language and use of interpretation. Providers may benefit from further instruction on properly collaborating with interpreters [14, 43] and on educating patients about building trust with interpreters [44], as interprofessional training for providers, interpreters, and translators has been shown to improve knowledge, confidence, practice, and attitudes towards collaboration [45]. Healthcare systems could implement training for healthcare professionals on cultural competency and language skills. They could prioritize efforts to audit and improve the quality of interpretation to address concerns raised by Spanish-preferring patients regarding misinformation and misunderstandings. Research could focus on evaluating the impact of language concordance on continuity of care, health outcomes, and patient engagement to inform evidence-based practices in primary care. Providers could also benefit from knowing that patients value continuity of providers and recognize the privilege in patient-provider relationships.

It has been more than twenty years since the propagation of the U.S. Department of Health and Human Services’ guidance on providing care to patients with limited English proficiency. Future guidance should account for the advances in the use of remote phone and video interpretation and growing comfort of patients (and providers) using telehealth due to the COVID-19 pandemic. Guidance could include recommendations to increase scheduled visit length when clinics know a patient needs interpretation. Federal policymakers could focus on implementing quality assurance measures for interpretation services to address concerns by patients regarding misunderstandings. Establishing standards for interpreter use, training, certification, and monitoring can help ensure accurate and effective communication between providers and patients. This may be even more important for alternative languages, where patients are even less likely to have a bilingual provider.

Spanish-preferring patient experiences highlighted the necessity for healthcare systems to support interpretation services that enhance direct communication, ensure interpreter quality, and maintain provider-patient relationships. Improvements in interpretation policy and practice are needed to optimize provider-patient communication, which is critical for high-quality health outcomes and experiences.

### New Contribution To the Literature

This study examines experiences of Spanish-preferring patients receiving care at two large, urban FQHCs comparing their experiences with providers who are language-concordant or who use formal or informal interpreters. Much of the prior research on this topic has relied on the perspectives of providers and interpreters; in this study, we spoke to patients directly. The study revealed that Spanish-preferring patients value continuity with their healthcare providers. However, experiences varied significantly depending on the type of language support provided, with Spanish concordant experiences focusing on multiple concerns albeit briefly, care using formal interpreters enhanced understanding but sometimes made interactions feel impersonal, and the use of informal interpreters leading to inconsistent communication quality.

## Supplementary Information

Below is the link to the electronic supplementary material.

Supplementary material 1 (DOCX 48.7 kb)

## Data Availability

Data sharing is not applicable for this article as confidentiality was given the interviewees who participated in the study.
