# Supplementary material for: Language Concordance and Interpreter Use in Primary Care: Perspectives from Spanish-preferring Patients
Source: J Immigr Minor Health. 2025 Sep 3;28(1):187–202. doi: 10.1007/s10903-025-01768-w (PMC12882946; doi:10.1007/s10903-025-01768-w)
Supplement: Supplementary file 1 — Supplementary material 1 (DOCX 48.7 kb) [file 10903_2025_1768_MOESM1_ESM.docx]

SUPPLEMENTAL MATERIALS

*Title:* Language Concordance and Interpreter Use in Primary Care: Perspectives from Spanish-preferring Patients

*Journal Name:* Journal of Immigrant and Minority Health

SUPPLEMENTAL METHODS

*Participants.* [BLINDED] received information from the FQHCs concerning the names, contact information, gender, date of last visit, and provider type (e.g., medical doctor, nurse practitioner, physician assistant) at last visit for all Spanish-preferring patients who had a recent face-to-face visit between September 15, 2022, and March 15, 2023 (FQHC#1: n=4,403, FQHC#2: n=718).

**Fig. 2** Sampling and Recruiting Flow Diagram


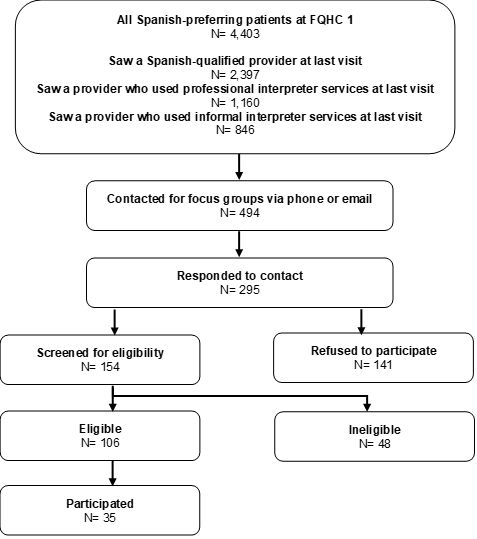


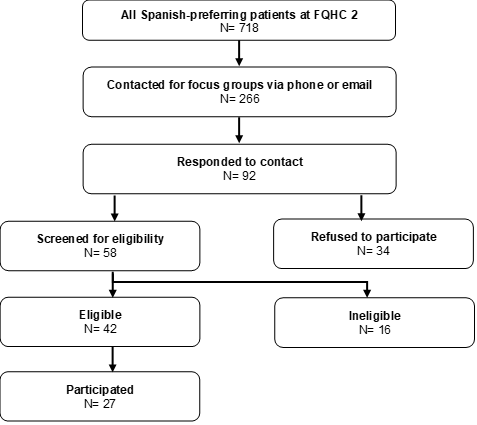


We stratified the data by provider type and created sampling queues with the aim of recruiting 6-8 participants per group evenly balanced by gender. We screened patients to determine whether at their last visit they were seen by a provider who spoke Spanish, a provider who used formal professional interpreters (via phone or video) or whether a provider used a strategy other than a formal interpreter.

Out of the 4,403 Spanish-preferring patients at FQHC#1, 2,397 were patients who at their last visit saw a Spanish-qualified provider, 1,160 patients saw a provider who used professional interpreter services (phone or video), and 846 patients saw a provider that used informal interpreter services. Of these, 494 were called or emailed, of those 295 responded with 141 who refused to participate and 154 were screened for eligibility, yielding 48 ineligible individuals. We identified 106 patients who were eligible to participate in a focus group and ultimately 35 participated from FQHC#1. Out of the 718 patients at FQHC#2, 266 were called or emailed, of those 92 responded with 34 who refused to participate and 58 were screened for eligibility, yielding 16 ineligible individuals. We identified 42 patients who were eligible to participate in a focus group and 27 participated from FQHC#2. Some eligible patients did not participate because they were not available for a specific focus group date, canceled 2 days prior, or were a no show (i.e., illness). We found no significant differences (p-value<0.05) by gender or by provider type across responders (vs non-responders), those who refused (and agreed to be screened), those who were eligible (vs not eligible) and those that participated (and did not participate) (data not shown).

*Provider and Clinic Staff Characteristics.* Many of the providers working at the FQHCs, including most of the front office staff and nurses, speak Spanish fluently. Some of the providers have also gone through the additional step of obtaining an official Spanish qualification; for those who have not, per the FQHCs’ policies, they must use the video interpretation services available in the clinic. At the time of the study, the first FQHC employed 301 providers, of whom 239 were primary care providers. Of these 239 providers, 104 served predominantly Spanish-preferring patients. Of the 104 providers serving Spanish-preferring patients, 21 were Spanish-qualified and 83 were not. Of the 83 providers, only 27 regularly used interpreter services, leaving 56 who mainly used other strategies for communication.

*Survey Data Collection*. Participants completed a 46-item paper survey with an open-ended question at the end prior to the focus group (upon arrival) about their care experiences at their last visit at the FQHC including questions about their interactions with providers, nurses, and interpreters (if applicable) and ratings of the provider, interpreter (if applicable) and clinic. The survey included CG-CAHPS Visit survey items on provider communication, overall provider rating, patient demographics, and CAHPS supplemental items on interpreter use, and items on the patient trust of the provider and nurse[46].

*Analysis.* We used t-tests to compare the case-mix adjusted means for the Spanish-preferring patient focus group responses across the three types of focus groups. We also conducted t-tests to compare the case-mix adjusted means of CG-CAHPS provider communication and overall provider rating for Spanish-preferring patients who attended the focus groups to all patients of all providers at the FQHCs during the same sampling timeframe.
